# Arthrogryposis Multiplex Congenita (AMC) and counselling before and during pregnancy: a questionnaire study

**DOI:** 10.1186/s13023-025-03913-y

**Published:** 2025-07-26

**Authors:** Arda Arduç, Julia Slootbeek, Johanna I. P. de Vries, Maria B. Tan-Sindhunata, Femke Stoelinga, Bonita Sawatzky, Isabel Filges, Ingeborg H. Linskens, for the Arthrogryposis and Pregnancy Study Group, Victoria Castillo, Carolina Navalon, Ani Samargian, Donna Davis, Remco Jansen

**Affiliations:** 1 Department of Obstetrics and Gynaecology, Amsterdam UMC, University of Amsterdam, Meibergdreef 9, 1105 AZ Amsterdam, the Netherlands; 2 Amsterdam Reproduction and Development, Amsterdam, The Netherlands; 3 Department of Human Genetics, Amsterdam UMC, University of Amsterdam, Amsterdam, the Netherlands; 4 Department of Rehabilitation Medicine, Amsterdam UMC, University of Amsterdam, Amsterdam, The Netherlands; 5 Member of the Adult AMC International Registry and Associate Professor, Department of Orthopedics, Faculty of Medicine, University of British Columbia, University Endowment Lands, Canada; 6 Institute of Medical Genetics and Pathology, University Hospital Basel and University of Basel, Basel, Switzerland

**Keywords:** Arthrogryposis Multiplex Congenital, Pregnancy, Counselling, Mode of delivery

## Abstract

**Background:**

Pre-pregnancy counselling in women with Arthrogryposis Multiplex Congenita (AMC) is not implemented as standard care. Prior surveys revealed that the majority of adults with AMC live independently with or without support, are working, and engage in social actifities. Although many of them underwent pregnancies, literature is scarce concerning women with AMC and pregnancies. This study enquires information of women with AMC and their preferences to optimize counselling and guidance concerning aspects related to (pre-)pregnancy, childbirth and parenthood. Women with AMC, being members of an international AMC patient support group or Canadian register, were invited anonymously via newsletter in mail or announcement on social media.

**Results:**

A total of 53 women with confirmed AMC participated in this questionnaire study. Pregnancies were reported in 64.2% (34/53) of the women and 26 women had multiple pregnancies and delivered in total 45 children. A third (15/45) of the children were born vaginally and the remaining per caesarean section. None of the children had AMC. Four women reported on complications during application of local analgesia in labour and in 3 while positioning on the operating table. Of the 47 women who answered this question, 95.7% (45/47) expressed a wish for standard pre-pregnancy counselling. As optimal circumstances they prefer counselling at or above 18 years of age, by a gynaecologist, at the outpatient clinic and for those with a relationship together with their partner. The importance of multidisciplinary team of physicians was emphasized as well as information from peer support groups and websites. Women expressed concerns about lack of knowledge on AMC in healthcare workers, discontinuity in care and attitude towards treatment and acknowledged the importance of this study.

**Conclusions:**

This study provides unique insights from women with AMC regarding pregnancy and parenthood. Participants emphasized the importance of tailored care. Their preferences strongly advocate for implementing pre-pregnancy counselling from the age of 18, ideally by a multidisciplinary team including a gynecologist. Incorporating these perspectives is essential to improve reproductive care.

**Supplementary Information:**

The online version contains supplementary material available at 10.1186/s13023-025-03913-y.

## Background

Pregnancy-related questions arise in women diagnosed with the rare condition Arthrogryposis Multiplex Congenita (AMC), which affects approximately 1 in 3000–5000 newborns 1. AMC is an umbrella term for conditions with heterogeneous phenotypes and underlying causes 1. These conditions are characterized by contractures (joints lacking full normal motion) in various anatomical regions 1, 2. The affected joints restrict movements, often cause physical impairment and chronic back or joint pain, resulting in limitations in daily functioning 1, 3. Pregnancy-related questions may emerge in individuals with AMC, a condition in which the majority live independently, pursue education or employment, and actively engage in social activities [4–8. Approximately half of the people with AMC manage this independency without assistance and the other half require extra aids such as power mobility or braces 3, 7.

Pre-pregnancy counselling is not routinely offered to women with AMC, despite the reported challenges in pregnancies with AMC, caused by immobility, associated anomalies (e.g. abnormal orofacial development) and occasionally impaired respiratory system 9. This is in contrast to other chronic medical conditions, such as diabetes, hypertension, and thyroid disease for which awareness already exists that optimizing the healthcare before and during pregnancy can reduce the risk of adverse effects for both mother and child 10, 11. In our recent scoping review, we provided an overview of key perinatal care aspects, along with additional recommendations, including potential anesthesiologic challenges that should be anticipated 12.

Currently, there is a lack of literature on pregnancy-specific challenges in women with AMC 2. Sawatzky et al. previously highlighted this gap using a scoping review and Delphi study to identify outcome measures for adults with AMC 2. Our recent scoping review further confirmed this knowledge gap, revealing only 27 published reports covering 82 pregnancies in 43 women with AMC, primarily with distal arthrogryposis 12. These two scoping reviews emphasized the need to understand not only clinical outcomes but also the informational needs and preferences of women with AMC regarding (pre-)pregnancy, childbirth, and parenthood.

This study aims to fill that gap by evaluating how to optimize counselling and guidance of women with AMC concerning aspects related to (pre-)pregnancy, childbirth, and parenthood, including the anticipated challenges of physical caregiving tasks, breastfeeding, and organizing support for newborn care, which may be affected by the functional limitations associated with AMC. The questionnaire encompasses the characteristics of the women with AMC (general and medical background, education, work, and social life), experience during earlier pregnancies, information before pregnancy, during pregnancy, delivery and after birth, as well as personal opinions and advices. The findings of this study will empower healthcare providers to implement personalized (pre-) pregnancy counselling and tailored healthcare that align with the preferences of women with AMC.

## Results

The study population includes 53 women with AMC. Initially, 71 women started this study. Seventeen women, however, answered the characteristics section alone and were therefore excluded from the result analysis. These 17 women were evenly distributed over the various countries, with similar age ranges and severity of AMC concerning affected joints compared to the remaining participants. Another woman was excluded due to not having the diagnosis of AMC herself, although her child did. Below the main aspects are highlighted per theme. The survey and all results of the survey are demonstrated in Additional file Tables 1–8.

## Theme 1. Characteristics

The median age in 5-year period of the women was 36–40 years (range 16–20 to 71–75 years). Participants reported their current country of residence: 40 out of 53 lived in Europe, 12 in North America, and 1 in New Zealand. Reported joint involvement, operations, and mobility impairment by AMC, confirmed that all women fulfilled the phenotypic description of presence of contractures in more than one anatomic region. Underlying cause of AMC was known in 14/53 (1 Amyoplasia, 6 genetic or non-genetic inheritable, and 7 environmental). Genetic tests have been performed in 19/53 (36%) women in the period 1980–2023. Living independently was reported by 62.3% of participants (33/53), including those living alone (n = 3), with a partner without children (n = 12), or with a partner and children (n = 18). The remaining women were living with parents or other family members in 22.6% (12/53), or with help from other people in 15.1% (8/53). Women were employed in 67.9% (36/53). Reasons not to work were being a student (n = 4), retirement (n = 5), having limitations by AMC (n = 5), taking care of children (n = 2), and searching for a job (n = 1). In 69.8% (37/53) women reported to have a live partner. Having energy for hobbies and sports, such as physical and creative activities was reported in 58.5% (31/53). The quality of life of the women with AMC concerning physical, mental and social functioning was scored with a median of 7 (range 1–10).

## Theme 2. Experience during earlier pregnancies

Pregnancies were reported in 34 of the 53 women (64.2%). From the 34 women with pregnancies, a total of 26 women gave birth to 45 children, who were healthy and unaffected by AMC. A third (15/45) of the children were born vaginally and the remaining per caesarean section. The majority of cesarean sections were planned: 18 out of 30 (60%) were scheduled early in the pregnancy, and 5 (16.7%) were planned closer to the time of delivery. Two cesarean sections were unplanned (6.7%), and 5 (6.7%) women could not recall the details. The reported experience during earlier pregnancies are listed in Table 1.

**Table 1:** Experience during earlier pregnancies of women with AMC, survey study in 2024

	Number/ completed questions (percentage)
*Ever been pregnant* Yes Yes and gave birth to alive child No, reasons: Own choice (*n* = 15); advice health care provider (*n* = 2); future wish to get pregnant (*n* = 1); not able to have intercourse (*n* = 1)	34/53 (64.2%) 26/53 (49.1%) 19/53 (35.8%)
*Total number of alive children* Number of (alive) children per woman 1 2 3 4 5	45 12/26 (46.2%) 11/26 (42.3%) 2/26 (7.7%) 0 1/26 (3.8%)
*Location of delivery* Hospital Midwife assisted Doctor assisted Midwife and doctor assisted	45/45 (100%) 3/26 (11.5%) 18/26 (69.2%) 5/26 (19.2)
*Vaginal delivery* Uneventful Difficulties application epidural/spinal Difficulties application iv drip Vacuum/ forcipal extraction	10/15 (66.7%) 2/15 (13.3%) 0/15 3/15 (20%)
*Caesarean section* Uneventful Difficulties application epidural/ spinal Difficulties application iv drip Difficulties position on operation table	27/30 (90%) 2/30 (6.7%) 0/30 3/30 (1%)
*The course of pregnancy lead to refrain from future pregnancies* No Yes Do not know yet	21/34 (61.8%) 12/34 (35.3%) 1/34 (2.9%)
*Change in mobility during or after pregnancy* Cause increase in pain Difficulties walking Worsening of joint complaints Complaints like fatigue and swelling of the legs	18/26 (69.2%) 7/18 8/18 4/18 4/18
*Gestational age at birth* Term birth Preterm birth Unknown	35/45 (77.8%) 7/45 (15.6%) 3/45 (6.7%)
*Extra help from own network (friends or family)* During pregnancy After pregnancy No extra help	14/34 (41.2%) 17/34 (50.0%) 13/34 (38.2%)
*Extra help for housekeeping/household* During pregnancy After pregnancy No extra help	12/34 (35.3%) 18/34 (52.9%) 15/34 (44.1%)
*More assistance required with self-care* During pregnancy After pregnancy No extra help	13/34 (38.2%) 11/34 (32.4%) 18/34 (52.9%)
*Adaptions/help for breastfeeding* Yes No	8/26 (30.8%) 18/26 (69.2%)
*Adaptions/help for formula feeding* Yes No	6/26 (23.1%) 20/26 (76.9%)
*Adaptions/help for childcare* Yes No	12/26 (46.2%) 14/26 (53.8%)

## Theme 3. Information before pregnancy

Participants talked with healthcare providers about the wish to have children in 57.7% (30/52), sexuality in 37.3% (19/51), and fertility in 49.0% (25/51). Women reported that they did talk with their own healthcare providers about the wish to have children, but the healthcare providers could not answer questions related to the three topics in 36.7% (11/30) child wish, 15.8% (3/19) sexuality, 20% (5/25) fertility, respectively. Women found it difficult to talk about these topics (21.2%; 35.3%; 19.6%) and healthcare providers had no time to talk about it (3.8%; 11.8%; 7.8%). Other women did not feel the need to talk about it with their healthcare providers (13.5%; 17.6%; 17.6%). Participants had an active sex life without pain [60.8% (31/51)] or with some extra pain [23.5% (12/51)]. Two women (aged 54–60 years) reported that sex was very painful. Both had contractures in the upper and lower limbs and had undergone multiple lower limb surgeries. They were independently mobile without assistive devices. There were no women with absent sex life due to pain.

The preferences concerning a pre-pregnancy counselling are shown in Table 2. The three other most preferred ways to be informed about pregnancy and AMC include: by other patients with AMC [59.6% (31/52)], internet or websites [54.8% (28/52)], and a meeting with presentations by healthcare providers [51.9 (27/52)]. Approximately half of the women [49.0% (25/51)] had ever heard of pre-pregnancy counselling. Most women [95.7% (45/47)] found pre-pregnancy counselling to be useful as standard care. In 73.6% (39/53) women expressed openness to genetic counselling regarding new possibilities in genetic testing. Curiosity [59.0% (23/39)] as well as family planning [35.9% (14/39)] are the main arguments for this.

**Table 2:** Preferences concerning information before pregnancy expressed by women with AMC, survey study in 2024

	Number/completed questions (percentage)
*Preferred age to get information about fertility and future child wish* 15–16 years old 17–18 years old > 18 years old Open answer: Age-appropriate information from nursery or primary school onwards Open answer: not specified	2/51 (3.9%) 4/51 (7.8%) 32/51 (62.7%) 2/51 (3.9%) 11/51 (21.6%)
*Preferred company during pre-pregnancy counselling* Alone (without anyone else) Together with my partner Together with my parents I don’t know Open answer: not specified	14/50 (28.0%) 26/50 (52.0%) 1/50 (2.0%) 1/50 (2.0%) 8/50 (16.0%)
*Preferred healthcare provider to give pre-pregnancy counselling* General practitioner Gynaecologist Anesthesiologist Rehabilitation doctor/physiatrist Midwife Neurologist Clinical geneticist Combination of doctors Open answer: trauma surgeon Open answer: not specified	17/50 (34.0%) 39/50 (78.0%) 9/50 (18.0%) 5/50 (10.0%) 13/50 (26.0%) 7/50 (14.0%) 20/50 (40.0%) 11/50 (22.0%) 2/50 (4.0%) 7/50 (14.0%)
*Preferred appointment for pre-pregnancy counselling* Outpatient clinic By phone (with or without seeing each other/video-interactive meeting) In hospital Open answer: At mutual insurance company Not specified	27/50 (54.0%) 11/50 (22.0%) 2/50 (4.0%) 1/50 (2.0%) 9/50 (18.0%)
*Preferred topics during pre-pregnancy counselling* Fertility Medication to be stopped before pregnancy Influence of pregnancy and parenthood on daily functioning with AMC Influence of AMC on baby’s and own health Most common difficulties during pregnancy in women with AMC Possibilities of genetic testing before/during pregnancy and chance for heredity Possibility of 20-week ultrasound during pregnancy to exclude AMC Information on labour and preparing “Birth plan” Breastfeeding and possibility of referral to occupational therapist for advice Possibilities to deploy help to maximize the independency around pregnancy Open answer: Emotions that may arise, maternal mental health Open answer: Guidance and public subsidies for IVF treatment	33/50 (66.0%) 29/50 (58.0%) 37/50 (74.0%) 37/50 (74.0%) 39/50 (78.0%) 37/50 (74.0%) 29/50 (58.0%) 35/50 (70.0%) 31/50 (62.0%) 32/50 (64.0%) 1/50 (2.0%) 1/50 (2.0%)

## Theme 4. Information about pregnancy, delivery and period after delivery

An overview of preferences for receiving information concerning pregnancy, birth plan and after delivery is depicted in Table 3. Women prefer to discuss their birth plan with their healthcare providers multiple times before and during the pregnancy. Thirty-three women (71.7%, 33/46) appreciate the possibility to have a separate consultation with an anesthesiologist. Eleven women (23.9%, 11/46) described that they had a consultation with an anesthesiologist during a prior pregnancy.

**Table 3:** Preferences concerning information about pregnancy, delivery and period after birth of women with AMC, survey study in 2024

	Number/ completed questions (percentage)
*Feel supported if during pregnancy gynaecologist collaborates with my general practitioner for AMC* Yes, early in pregnancy Yes, before the delivery No Open answer: Don’t have a doctor for AMC	26/47 (55.3%) 18/47 (38.3%) 11/47 (23.4%) 1/47 (2.1%)
*Expected healthcare provider(s) to be in need during pregnancy (other than obstetric caregiver)* General practitioner Rehabilitation doctor Neurologist Orthopaedic surgeon Social worker Psychologist Anesthesiologist Clinical geneticist Paediatrician Multidisciplinary team with tailored care for own situation with AMC Open answer: Occupational therapist	25/47 (53.2%) 18/47 (38.3%) 7/47 (14.9%) 11/47 (23.4%) 7/47 (14.9%) 10/47 (21.3%) 23/47 (48.9%) 18/47 (38.3%) 18/47 (38.3%) 18/47 (38.3%) 1/47 (2.1%)
*Topics birth plan* Location of labour Mode of delivery: spontaneous vaginal delivery Mode of delivery: induced labour Mode of delivery: caesarean section Discussion about at which pregnancy week Possibility for pain relief Which healthcare provider present during labour If vaginal delivery: position during delivery Breast and formula feeding Extra care after birth	29/46 (63.0%) 21/46 (45.7%) 15/46 (32.6%) 31/46 (67.4%) 22/46 (47.8%) 31/46 (67.4%) 28/46 (60.9%) 25/46 (54.3%) 30/46 (65.2%) 36/46 (78.3%)
*Preferred mode of delivery adapted to your AMC* Vaginally Caesarean section No preference, but would prefer to make choice during labour Not applicable, don’t have the wish to become pregnant Open answers: No preference, but would prefer to discuss the options in consultation and receive information about what is possible and can be facilitated (e.g. adaption of delivery table)	12/46 (26.1%) 19/46 (41.3%) 7/46 (15.2%) 4/46 (8.7%) 4/46 (8.7%)
*Expect to need extra support for*	**Agree (number/ completed questions (percentage))**
Physical care of myself and child from own network after dellivery	33/46 (71.7%)
Households after delivery	41/46 (89.1%)
Breastfeeding after delivery	24/46 (52.2%)
Formula feeding	23/46 (50.0%)
Care for child	26/46 (56.5%)

## Theme 5. Information about period after birth

The expected extra help or aids after birth are presented in Table 3.

## Theme 6. Opinions and advices

Reported opinions, ideas and advice are categorized for healthcare, knowledge on AMC and organization aspects and summarized concerning all 53 women in Table 4. Advice around pregnancy given by the women with a prior pregnancy are categorized for knowledge on AMC and suggestions for support and presented in Table 5. To illustrate concerns of women with AMC, we cite below from the free text answers, and all individual answers are listed in Additional File Table 9.

**Table 4:** Ideas for ideal pre-pregnancy counselling, experienced and suggestions by women with AMC, survey study in 2024

Ideas for ideal pre-pregnancy counselling (*n* = 44)	
With history of pregnancy (*n* = 31)	Without history of pregnancy (*n* = 13)
Healthcare provider Patient tailored information by most frequent visited doctor (*n* = 1) Concern about knowledge of gynaecologist (*n* = 1) * Tailored specialist: first GP and afterwards gynaecologist (*n* = 1)	Healthcare provider Tailored specialist: first GP and afterwards gynaecologist (*n* = 2)
Knowledge Doctor with knowledge of AMC (*n* = 4) More academic research on AMC in general (*n* = 1)	Knowledge Availability of an updated list of genes involved in AMC (*n* = 1)
Organisation During teens (*n* = 1) Age 18 + (*n* = 1) Peer contact with fellow AMC’er that experienced pregnancy (was not available during own pregnancy) (*n* = 1) Online counselling (*n* = 2) “In a place that builds trust” (*n* = 1) Deciding when you want to have children (*n* = 1) Multidisciplinary approach (*n* = 2) Need for counselling, since experienced current absence (*n* = 1) Organisation of care for patient self and child (*n* = 2)	Organisation Personalized care: awareness is asked for over treating / undertreating / “ableism” (*n* = 2)*
Other topics Limitations (*n* = 1) Exploring possible emotions that arise after birth (*n* = 1) Expectations (*n* = 1) Fertility, inherit, risks (*n* = 1)	Other topics Consequences of weight gain during pregnancy (*n* = 1)
No additional ideas (n = 15)	No additional ideas (n = 8)
Expressing contentment towards the study (n = 2)	
**Opinions, experiences, and suggestions (*n* = 25)**	
**With history of pregnancy (*n* = 17)**	**Without history of pregnancy (*n* = 8)**
Experiences with organisation Consultant referrals were made but not realised (*n* = 1)* Referred for counselling but in absence of knowledge no counselling was realized (*n* = 1) Delivery room areas and bathroom not accessible (*n* = 1)* Caesarean prevented visiting her children in incubator for 24 h after birth (*n* = 1)	Experiences with organisation Wish for peer contact (*n* = 1) Refusal of pre-pregnancy counselling (*n* = 1) Lack of educated doctors on AMC and pregnancy (*n* = 1)*
Opinions Contacting a geneticist who specialized in AMC was very helpful (*n* = 1) Website lactancia.org very useful (*n* = 1)	Opinions None
Suggestions Multidisciplinary team, adaptions and recourses for people with reduced mobility. Training and information for health professionals (*n* = 1)	Suggestions For future research: differentiate between major and minor surgeries (*n* = 1) Increase of awareness for the need of pre-pregnancy counselling (*n* = 1)
Requesting information sources (*n* = 3) a.o.: wish for online or research forums	Requesting information sources and tools (*n* = 2)
Expressing gratitude and contentment towards the study (*n* = 9)	Expressing gratitude and contentment towards the study (*n* = 2)
No additional comments (*n* = 2)	No additional comments (*n* = 2)

^*^Elaboration in form of a quote in the text (Results theme 6)

**Table 5:** Advices given by 31 women with AMC and experienced pregnancies, survey study in 2024

To women with AMC	To healthcare providers
Knowledge on AMC and pregnancy	
Seek counselling and preparation for childbirth and breastfeeding, ask everything (without feeling burdened) (*n* = 5) a.o.: reach out to someone who has been there, midwife workshops, mode of delivery: “Having hip problems is not synonymous with having to give birth by caesarean section”* but also “Do not try vaginally, just go for the caesarean section”	More knowledge of AMC amongst treating doctors (*n* = 5) a.o.: to prevent the need for repeating explaining, to prevent anxiety among healthcare providers during treatment, more knowledge on challenges with intravenous line insertion, mobility problems after pregnancy, delay in recovery after CS
Follow research updates on AMC (and baby movement) (*n* = 1)	More knowledge on physical disabilities and understanding of patients moving differently, experienced as safe (*n* = 4) a.o.: training and preparation for healthcare personnel to care for patients with physical disabilities / AMC (*n* = 2)
	Assist in finding way to hold, breastfeed/formula feed, and change child(ren) (*n* = 1) *
	Inform patient of limitations and problems (*n* = 2)
*Supportive help/treatment*	
Look for a gynaecologist that is supportive and inspiring (*n* = 1)	Personalized care: awareness is asked for overtreating / undertreating / “ableism” (*n* = 8) a.o: balance between care and intruding in bonding time, do not judge someone on their ability to carry a child because of the physical disability, do not overdo it, allow relaxation, “More humanity” (*n* = 3)
Ask for and accept any extra assistance from friends, family and professionals (*n* = 2)	Diagnostics: additional ultrasounds, prior genetic counselling, (free) genetic testing (*n* = 7)
Try gentle exercising, such as swimming (*n* = 3), control weight as much as possible (*n* = 1)	Facilities: accessibility in offices, delivery room and maternity ward, privacy for extra assistance in private room if possible (*n* = 3)
	Active support on mental wellbeing and experienced care (*n* = 1)
Enjoy the pregnancy and your children, live the precious experience (*n* = 3)	Listen to your patient, she knows her body better than anyone (*n* = 2)
Expressing contentment towards their pregnancy and childbirth (n = 2)	
No additional advices (*n* = 5) a.o since miscarriage in first trimester (*n* = 1)	

^*^Elaboration in form of a quote in the text (Results theme 6)

One participant with one vaginal birth and one caesarean, and history of surgery on the hips, knees and feet mentioned, “Having hip problems is not synonymous with having to give birth by caesarean section.”

A woman that chose not to have a child partially due to lack of educated doctors around AMC and fearful of how her body would adapt to pregnancy. “Women with AMC need to be given the same counselling and information about pregnancy and childbirth as able bodied women. The medical field has a lot of internalized ableism around this and seldom initiated conversations about this with me.”—With better resources, I may have chosen differently”.

A few participants shared their experience of feeling unsupported during her pregnancy despite early efforts to arrange care. They described difficulties in accessing referred specialists and a lack of communication with the healthcare provider, which left them uncertain about how her pregnancy would progress.

Another comment was about the importance of assistance in finding a way to hold, breastfeed and formula feed the child(ren) “This can mean using feet to change a diaper, or even the mouth, using the mouth or chin to lift and position the baby or the need to have our kids placed on/next to us by our partners or personal assistants”.

A further remark was given on the well-prepared organization during pregnancy “It was helpful to have additional ultrasounds to identify if there were any contractures. Not enough gynecologists and obstetricians have accessible offices and my OB expressed anxiety over treating me due to complications. The anesthesiologist was fantastic and was prepared for my needs”.

## Discussion

This study focused on the preferences of an international cohort of 53 women with AMC on pregnancy-related topics. The majority of women experienced a normal sex life, two third became pregnant and half of the women delivered children. These findings emphasize the importance of the focus on possibilities of pregnancy and parenthood in AMC. Their experiences and advices regarding standard implementation of (pre-)pregnancy counselling and experiences around pregnancy care are explicit.

This population of women with AMC was reached through patient support groups and one AMC register. The underlying cause in this population was found in about a quarter of the women. While prior studies report that Amyoplasia represents about one-third of AMC, in this study only one woman (2%) had Amyoplasia 1. Furthermore, a genetic or non-genetic heritable cause was found in 6 women (31%), which is also lower than the genetic yield that was reported in prior studies with individuals with AMC (up to 52.7–65.2%) 13, 14. However, the lower genetic yield may be partially explained by the fact that genetic testing in many participants was performed before the widespread clinical implementation of advanced sequencing techniques such as next generation sequencing based tests (whole exome sequencing) 15. Educational level, work, and quality of life experience of the participants are in line with prior studies 3, 16. Studies of Altiok and Nouraei showed also a high quality of life among individuals with AMC 3, 16. The level of independency of the participants was also similar to previous quality of life and AMC studies (51.8–75%) 3, 8.

Pregnancies were reported in two third of this study population and half delivered children of which one third vaginally and two third per caesarean sections. While this caesarean rate is higher than in the general population, it aligns with findings in other populations of women with neuromuscular disorders or physical disabilities 17. Awater et al. reported similarly elevated caesarean rates in a cohort of 178 women with hereditary neuromuscular disorders, primarily due to obstetric indications such as abnormal fetal presentation, concerns about maternal muscle function, or pelvic anatomy 17. The deliveries were uneventful in two third of the vaginal deliveries and in 90% of the caesarean sections. A few had applications problems with the epidural during labour and none with introduction of intravenous drip. The fact that the majority of cesarean sections were planned suggests they were performed due to maternal indications. In this population, none of the born children had AMC.

The received information before pregnancy concerning pregnancy-related aspects has been experienced as insufficient among the participants. Half of the women had heard of the possibility of pre-pregnancy counseling, but nearly all would (have) preferred it as a standard procedure. The lack of doctor-patient communication concerning sexuality and fertility were mentioned by the participants. This is in line with other reports on women with a physical impairment who experience that healthcare providers pay limited attention to sexual activity, and fail to inquire about the effect of the impairment on sexuality and the wish to get pregnant 7, 18. Healthcare providers might feel ambivalence and discomfort to discuss these topics 19, 20. Absence of such information prevents some women to not fulfil their wish to have children 21. In our study, this was also reported by one of the participants. The most optimal implementation of pre-pregnancy counselling was considered to be performed above 18 years of age, provided by a gynaecologist, at the outpatient clinic, and together with their partner. It is important to know that the majority of women with AMC is interested to be counseled again about new possibilities for genetic testing, such as preimplantation genetic testing.

During pregnancy, women prefer to receive information and care by a multidisciplinary team. This team will be tailored per woman with AMC. Moreover, half of the women expected care of a general practitioner and anesthesiologist in addition to a gynaecologist. A third of the women who had a prior pregnancy reported that they had been referred for consultation to an anesthesiologist. One women emphasized the safety that was felt when having a well-prepared anesthesiologist with knowledge of AMC. Given the potential for anaesthetic difficulties, there was a strong desire for thorough preparation 9. The majority of topics in the birth plan for timely preparation of the delivery were considered as important, but the main topic was to discuss the possible extra care needed after birth (78%).

During pregnancy, the increased body weight and growing uterus may affect posture and balance, increase joint strain, and can lead to fatigue. Although physical proportions generally normalize within the postpartum period (6–8 weeks after delivery), persistent fatigue related to circulatory and hormonal adaptations may delay musculoskeletal recovery. After birth, the majority of the women in this study expected to need help of their own network for physical care of themselves and their child, and for the households. However, our study showed that approximately half of the women who had delivered indeed needed extra help after the pregnancy with physical care or housekeeping and even less for self-care, breastfeeding, and formula feeding. Functional limitations associated with AMC may affect the ability to perform early parenting tasks independently. Some women described inventive adaptations, while others highlighted the essential role of partners or support networks in day-to-day infant care. These findings emphasize the need to include practical parenting support and planning as a formal part of pre-pregnancy and postpartum counselling for women with AMC. Addressing these challenges proactively may empower women and reduce anxiety during the early stages of parenthood.

With the obtained knowledge on the possibilities to deliver vaginally or per caesarean section we can improve our counselling. Women with AMC have to be aware of a higher chance of operative delivery in comparison to the global average of a general population, two third versus one third (30–32%) 22. Specific patient oriented preparation with an understanding of AMC has to be made.

During the counselling before and during pregnancy, the challenge on immobility by increased pain during pregnancy in nearly 70% has to be discussed. This pain will be primarily due to the physiological changes of the musculoskeletal system adapting to the growing uterine content 23. In case of advancing immobility, there is a risk factor for thromboembolism, and it should be evaluated if anticoagulation medication is needed 24.

The individual remarks concerning pregnancy-related topics revealed information which was not derived from the multiple choice questions. Some women expressed their concern about the knowledge on AMC by healthcare providers. They also gave examples illustrating lack of knowledge caused by not reading the medical chart prior to the consultation, by no communication from one healthcare to another. Simply reading of the charts by the healthcare provider prior to a visit is suggested to reduce the burden for women with AMC preventing to repeat their long and well known history at each visit. Several examples were given to illustrate experienced discrimination because of the physical challenges, one woman explicitly expressed her concern of ableism. The definition of ableism is persistent disability-related discrimination, which often results in psychological disadvantages for people with disabilities 25. Women with AMC emphasize the importance to evaluate opportunities and not just obstacles during pre-pregnancy counselling and care during and after pregnancy. The balance between facilitating adequate care, and prevent over- and undertreatment is delicate. Women with AMC would appreciate more familiarity with pregnancy in women with disabilities to improve easiness in posing open pregnancy-related questions without prejudice and in a wheelchair accessible setting. In terms of perceived satisfaction with healthcare and counselling, women with physical disabilities -including those with spinal cord injury or cerebral palsy- have expressed similar concerns 26, 27.

One of the strengths of this study is the focus on the preferences and ideas concerning pre-pregnancy counselling provided by women with AMC. The collaboration with patient support groups and one AMC register from nine countries in three continents facilitated this study. The clear answers on the many topics on pregnancy-related questions paves the way to arm healthcare givers and women with AMC with new information.

Despite valuable insights from our study, we must acknowledge its limitations. The study population is still limited. We cannot exclude recall and selection bias. However, we did not question specific details on for example medication use, but essentials amongst others number of pregnancies and gestational age at birth in a population with a median age of 36–40 years. Moreover, the open questions gave a wealth of information of individual experiences.

The findings suggest several implications for clinical practice, including the importance of implementing standard pre-pregnancy counselling, providing information through a multidisciplinary team, and ensuring healthcare providers are adequately informed about AMC-specific challenges, such as anesthetic risks, physical limitations, and postpartum care needs. While the preferences reported in this study were not analyzed by region, it is important to acknowledge that healthcare experiences may vary based on healthcare system structure, access to services, and cultural expectations. Future research should aim to explore these differences systematically, ideally through large, international registries that combine patient-reported experiences with verified clinical and genetic data. Such registries would enable meaningful subgroup analyses- by AMC subtype, severity, or healthcare system- and guide the development of evidence- based guidelines for reproductive care in women with AMC. We attributed to a Delphi method among medical professionals and people with AMC experience to define common data elements which should be recorded in the international register on AMC 28. Comparative studies including other groups of women with chronic illnesses or disabilities may also provide valuable insights into shared needs and gaps in current care models. Ultimately, these efforts can help bridge the knowledge and practice gaps and promote equitable, person-centered care for this underrepresented population.

## Conclusion

Women with AMC reported their experience with pregnancies and specific wishes to be informed about pregnancy-related topics. A lack of information on pregnancy-related topics was experienced and their wish is to implement pre-pregnancy counselling as standard care, preferable at the age of 18 years or older, by a gynaecologist in collaboration with a clinical geneticist and general practitioner, at the outpatient clinic, and with their partner. Women with AMC acknowledged the importance of this study. The close collaboration with the patient support groups and registers will further support future research for increasing knowledge among healthcare providers and implementation of standard pre-pregnancy counselling.

## Methods

### Study design

A questionnaire was initially developed by the researchers (A. Arduç and J.I.P. de Vries) and adapted after comments of the co-investigators, including the representatives of the patient support groups (Spain, the Netherlands, United Kingdom and United States of America) and the coordinator of the Canadian adult AMC register. In line with recommendations of other AMC questionnaire studies, questions are kept as accessible as possible by formulating short questions in laymen’s terms, using a positive approach concerning pregnancy and ability with AMC, and limiting duration to about 45 min to complete 2, 29. The distribution of questions per six themes was: (1) 2 questions on general background, 16 on medical background, 6 on social life, education and work; (2) 14 on experience during earlier pregnancies; (3) 14 on information before pregnancy; (4) 8 on information about pregnancy and delivery, including topics for the birth plan; (5) 5 on information about period after birth, and (6) 1 open question to share opinions and advices (Questions are presented in Additional File Table 1).

### Research ethics

The Medical Ethics Committee of the Amsterdam UMC agreed upon the questionnaire study and assessed that this study is not subject to the Medical Research Involving Human Subjects Act (2022.0579).

### Data collection

Women with AMC who are members of the patient support groups for AMC (including the Netherlands (www.spierziekten.nl), Spain (www.artrogriposis.org), United Kingdom (https://www.arthrogryposis.co.uk), and United States of America (www.amcsupport.org)) and the Canadian register (Adult AMC International Registry, B. Sawatzky, M.D., Ph.D.) were invited to fill in the questionnaire. Female members with AMC were approached via an email and/or an announcement on the social media of the patient support groups, or via an email by the Canadian register. The email and announcement contained information of the study and a link to the questionnaire. It was not traceable for the patient support groups, register, or researchers which women chose to participate in the study. The anonymous questionnaire was available in Dutch, English, Spanish, and French via the secured website Castor (www.castoredc.com). Data collection was conducted from April to December 2023.

### Study population

The study population included women with AMC of 16 years or older, who were invited by one of the before mentioned patient support groups or Canadian register. Exclusion criteria were not being able to read Dutch, English, Spanish, or French. The population size was calculated to become approximately 70 women with AMC, as we expected that we could reach out to 220 women with AMC through the European patient support groups, and that a third of them would answer. The latter is in line with 34% completion of a questionnaire by people with AMC from the English patient support group 6.

Despite the fact that the AMC diagnosis was not derived from medical charts, the AMC could be phenotypically confirmed in all women by combining information of three questions concerning involvement of two or more anatomical regions, functional abilities and operations.

### Statistical analysis

The collected data was transferred and organized in Excel, Microsoft 356 A3 for faculty. Descriptive analysis was performed using Excel. Data was presented as numbers with percentages and medians and ranges. Answers were adapted to the number of participants that responded to each question, as number of answers/total answers. The survey design permitted non-mandatory responses, leading to minor variations in the response denominators across different questions. The survey design permitted non-mandatory responses, leading to minor variations in the response denominators across different questions. All free text field data was manually categorized.

## Supplementary Information

Additional file 1.

Additional file 2.

## Abbreviations

AMC: Arthrogryposis multiplex congenita
FADS: Fetal akinesia deformation sequence
M.D.: Doctor of medicine
OB/GYN: Obstetrician–gynecologist
Ph.D.: Doctor of philosophy
UMC: University Medical Centers

## Declarations

### Data availability

The datasets used and/or analysed during the current study are available from the corresponding author on reasonable request. The questions and outcome of the survey are also available in the Additional File Tables 1–8.
