# Supplementary material for: Arthrogryposis Multiplex Congenita (AMC) and counselling before and during pregnancy: a questionnaire study
Source: Orphanet J Rare Dis. 2025 Jul 26;20:378. doi: 10.1186/s13023-025-03913-y (PMC12297761; doi:10.1186/s13023-025-03913-y)
Supplement: Supplementary file 1 — Additional file 1. [file 13023_2025_3913_MOESM1_ESM.docx]

**Additional File Table 1. Questionnaire Arthrogryposis Multiplex Congenita (AMC) and Pregnancy, survey study among women with AMC 2024**

**General background**

- 1. What is your age?
- 16-20 years old
- 21-25 years old
- 26-30 years old
- 31-35 years old
- 36-40 years old
- 41-45 years old
- 46-50 years old
- 51-55 years old
- 56-60 years old
- 61-65 years old
- 66-70 years old
- 71-75 years old
- 76-80 years old
- 81+ years old
  1. Country of living?
- The Netherlands
- France
- Spain
- Switzerland
- United Kingdom
- United States
- Canada
- Other: … (open answer possible)

**Medical background**

2.1 The joint contractures caused by AMC are located in? (multiple answers possible)

- - Upper limb(s)
  - Lower limb(s)
  - Spine and/or neck (inclusive scoliosis, a curvature in the spine)
  - Jaw
  - Other: … (open answer possible)
  1. Describe your independency concerning the use of the lower limbs
  - Walking independently without devices
  - Walking independently with braces
  - Walking partly independently, partly with aid(s), such as sticks/crutches/canes/walker
  - Walking partly independently, partly with wheelchair
  - Mainly with wheelchair
  - Other: … (open answer possible)

2.3 Describe your independency concerning your arms for daily activities such as dressing and physical care

- - Independent
  - Fully dependent
  - Partially independent (in need of some assistance)

2.4 Describe your independency concerning your arms for daily activities such as eating

- - Independent
  - Fully dependent
  - Partially independent (in need of some assistance)

2.5 Describe your independency concerning your arms for daily activities such as housekeeping/household (for example cleaning)

- - Independent
  - Fully dependent
  - Partially independent (in need of some assistance)
  1. Describe your independency concerning your arms for daily activities such as using a computer
  - Independent
  - Fully dependent
  - Partially independent (in need of some assistance)

2.7 Describe your independency concerning your arms for daily activities such as cycling

- - Independent
  - Fully dependent
  - Partially independent (in need of some assistance)

2.8 Describe your independency concerning your arms for daily activities such as driving

- - Independent
  - Fully dependent
  - Partially independent (in need of some assistance)
  1. Did you have previous surgery in your upper limbs (from shoulders to fingers)?
  - Yes
  - No

2.9.1 If yes, in which area(s) of the upper limbs?

- - Shoulder(s)
  - Elbow(s)
  - Wrist(s)
  - Finger(s)

2.9.2 If yes, note the number of operations of the upper limbs: ... time(s) (open answer possible)

2.10 Did you have previous surgery in your lower limbs (from hips to feet)?

- - Yes
  - No

2.10.1 If yes, in which area(s) of the lower limbs?

- - Hip(s)
  - Knee(s)
  - Ankle(s)
  - Feet
    1. If yes, note the number of operations of the lower limbs: … time(s) (open answer possible)

2.11 Did you have previous surgery in your spine and/or neck?

- - Yes
  - No
    1. If yes, note the number of operations of spine and/or neck: … time(s) (open answer possible)

2.12 Pain caused by AMC can be of important influence on your daily activities. Rate on a scale of 1 (no influence) – 10 (very much influence): … (gliding scale)

2.13 Fatigue caused by AMC can be of important influence on your daily activities. Rate on a scale of 1 (no influence) – 10 (very much influence): … (gliding scale)

- 1. Do you know what the underlying cause of AMC is in your situation?
  - Yes
  - No

2.14.1 If yes, what is the cause?: … (open answer possible)

2.14.2 If yes, is there a genetic cause?

- - Yes
  - No
  - I don’t know

2.15 Have you ever had any genetic tests to find the underlying cause of AMC?

- - Yes
  - No
  - I don’t know
    1. If yes, in which year(s) were these genetic test(s) performed?: … (open answer possible)

2.16 Possibilities for genetic testing increased during the last 10 years. If your genetic tests revealed no genetic cause in the past, there is the possibility to get counselling (again) about new genetic tests.

- - Yes, I would like to be informed about possible new genetic tests
  - No, I don’t wish to be informed about possible net genetic tests
  - I don’t know

2.16.1 If yes, please explain why you want to be informed about possible new genetic test? … (open answer possible)

2.16.2 If no, please explain why you don’t want to be informed about possible new genetic test? … (open answer possible)

**Social life, education and work**

- 1. How is your living situation at this moment?
  - Independent, on my own
  - Independent, with partner without children
  - Independent with partner and children
  - Living with parents
  - Living with help from other people (daily frequent support for daily care)
  - Living in a nursing home
  - Other: … (open answer possible)

3.2 What is your highest level of education?

- - Primary/elementary school
  - Secondary/high school
  - Vocational education or similar training
  - Bachelor’s Degree
  - Master’s Degree
  - Other: … (open answer possible)

3.3 Are you working?

- - Yes
  - No

3.3.1 If yes, are you working parttime or fulltime?

- - Parttime
  - Fulltime

3.3.2 If yes, paid work of volunteer (unpaid) work?

- - Paid work
  - Unpaid work/volunteer

3.3.3 If yes, what is your job?: … (open answer possible)

3.3.4 If no, you answered that you’re not working. Why not?

- - Because I’m a student
  - Because I’m retired
  - Other: … (open answer possible)
  1. Do you have a partner?
  - Yes
  - No

3.5 Do you have energy for hobbies and sports?

- - Yes
  - No
  - Not at this moment, but in the past I had energy for hobbies/sports
    1. If yes, what are your hobbies/sports?: … (open answer possible)

3.6 Quality of life is an indication how satisfied you are with your physical, mental and social functioning. Please indicate how you rate your quality of life in relation with AMC (1 = totally not satisfied – 10 = totally satisfied): … (open answer possible)

**Experience during earlier pregnancies**

- 1. Have you ever been pregnant (including miscarriages, termination of pregnancies and/or stillbirths)?
  - No, I have never been pregnant
  - Yes, I have been pregnant

4.1.1 If no, what was the reason that you have never been pregnant? (multiple answers possible)

- - It was my own choice not to get pregnant
  - This was on the advice of my healthcare provider (for example rehabilitation doctor/orthopaedic surgeon)
  - I had the wish to get pregnant, but I never had a full-term pregnancy
  - I was not able to be have intercourse
  - I was not able to use insemination support
  - Other: … (open answer possible)

4.1.2 If yes, how many times have you been pregnant? … time(s) (open answer possible)

4.1.3 If yes, how many alive children did you give birth to? … child(ren) (open answer possible)

4.1.3.1 If greater than 0 children, was/were one or more child(ren) healthy?

- - Yes
  - No

4.1.3.2 If greater than 0 children, was/were one or more child(ren) affected with AMC?

- - Yes
  - No

4.1.3.3 If greater than 0 children, was/were one or more child(ren) affected with other diseases?

- - Yes
  - No

4.1.4 If greater than 0 children, what was the mode of delivery of these child(ren)? (multiple answers possible)

- - Vaginal
  - Caesarean section (C-section)

4.1.4.1 If vaginal, how many vaginal births? … birth(s) (open answer possible)

4.1.4.2 If vaginal, per vaginal birth: at which weeks? … (open answer possible)

4.1.4.3 If vaginal, in case you had a vaginal delivery: (multiple answers possible)

- - It went uneventful without complications
  - The birth was assisted with a vacuum cup
  - The birth was assisted with a forceps
  - Difficulties occurred during inserting an intravenous drip
  - Difficulties occurred during application of local analgesia (epidural)
  - Other: … (open answer possible)

4.1.4.4 If caesarean section (C-section), how many caesarean sections? … c-sections (open answer possible)

4.1.4.5 If caesarean section (C-section), per caesarean section: at which weeks? … (open answer possible)

4.1.4.6 If caesarean section (C-section), when was/were the caesarean section(s) planned?

- - Yes, early in pregnancy
  - Yes, before the delivery
  - No
  - Other: … (open answer possible)

4.1.4.7 If caesarean section (C-section), in case you had a caesarean section

- - There were no complications
  - There were difficulties/complications during the caesarean section(s)

4.1.4.7.1 If yes to complications, which difficulties/complications occurred during your caesarean section(s)? (multiple answers possible)

- - Inserting an intravenous drip
  - Application of local analgesia (epidural or spinal)
  - Application of breathing tube (intubation)
  - Other: … (open answer possible)

4.1.5 If greater than 0 children, in case you delivered one or more children: where did you deliver? (multiple answers possible)

- - In an hospital (midwife assisted)
  - In an hospital (doctor assisted)
  - At home (midwife or general practitioner assisted)
  - Other: … (open answer possible)

4.1.6 If yes to question 4.1, did the course of your pregnancy lead to the decision not to become pregnant again?: … (open answer possible)

4.1.7 If yes to question 4.1, did you have extra help from own network (friends and family) during or after your pregnancy? (multiple answers possible)

- - During pregnancy
  - After pregnancy
  - No extra help

4.1.8 If yes to question 4.1, did you have extra help for housekeeping/household during or after your pregnancy? (multiple answers possible)

- - During pregnancy
  - After pregnancy
  - No extra help

4.1.9 If yes to question 4.1, did you have extra help with self-care during or after your pregnancy? (multiple answers possible)

- - During pregnancy
  - After pregnancy
  - No extra help

4.1.10 If greater than 0 children, did you have adaptations/help for breastfeeding after your pregnancy?

- - Yes
  - No

4.1.11 If greater than 0 children, did you have adaptations/help for formula feeding after your pregnancy?

- - Yes
  - No

4.1.12 If greater than 0 children, did you have adaptations/help for childcare (for example cleaning) after your pregnancy?

- - Yes
  - No

4.1.13 If greater than 0 children, did your mobility change during the pregnancy or after the labour?

- - No
  - Yes: … (open answer possible)

4.1.14 If yes to question 4.1, with your experience on pregnancy with AMC, what advice can you give the professional medical doctors and women with AMC to optimize care around pregnancy? Please explain: … (open answer possible)

**Information before pregnancy**

5.1 How would you prefer to be informed about pregnancy and AMC? Information by: (multiple answers possible)

- - Patient support groups
  - Information leaflets
  - Internet/website
  - Discussion forum on the internet
  - Other patients with AMC
  - Meeting with presentations by healthcare providers
  - Magazines
  - My own healthcare providers by an advice consulation
  - I don’t want to be informed about pregnancy-related topics and AMC
  - Other: … (open answer possible)

5.2 Did you talk with your own healthcare providers about the wish to have children?

- - Yes, I did
  - Yes, but my healthcare provider(s) couldn’t answer my questions about this topic
  - No, because healthcare providers never had time to talk about this topic
  - No, because I feel/felt like it’s difficult to talk about this topic
  - Other: … (open answer possible)

5.3 Did you talk with your healthcare providers about sexuality? (multiple answers possible)

- - Yes, I did
  - Yes, but my healthcare provider(s) couldn’t answer my questions about this topic
  - No, because healthcare providers never had time to talk about this topic
  - No, because I feel/felt like it’s difficult to talk about this topic
  - Other: … (open answer possible)

5.4 How is/was your sex life (in the period that you could become pregnant)? (multiple answers possible)

- - My sex life is/was normal and caused/caused no extra pain
  - My sex life is/was normal but caused/caused some extra pain
  - My sex life is/was nearly normal but is/was very painful
  - My sex life is/was severely restricted by pain
  - My sex life is/was nearly absent because of pain
  - Pain prevents any sex life at all
  - Not applicable

5.5 Did you talk with your healthcare providers about fertility?

- - Yes, I did
  - Yes, but my healthcare provider(s) couldn’t answer my questions about this topic
  - No, because healthcare providers never had time to talk about this topic
  - No, because I feel/felt like it’s difficult to talk about this topic
  - Other: … (open answer possible)

5.6 It’s possible to be advised about the wish to have children with a gynaecologist prior to a pregnancy. This is called a pre-pregnancy counselling. Have you ever heard of this?

- - Yes
  - No

5.7 At which age would you like to get information about fertility and future childwish?

- - 15-16 years old
  - 17-18 years old
  - >18 years old
  - Other: … (open answer possible)

5.8 With whom would you prefer to receive information about the wish to have children and pregnancy with AMC?

- - Alone (without anyone else)
  - Together with my partner
  - Together with my parents
  - I don’t know
  - Other: … (open answer possible)

5.9 From which of the following healthcare providers would you prefer to receive information about pregnancy and AMC? (multiple answers possible)

- - General practitioner
  - Gynaecologist
  - Anesthesiologist
  - Rehabilitation doctor
  - Midwife
  - Neurologist
  - Clinical geneticist
  - Combination of doctors: … (open answer possible)
  - Other: … (open answer possible)

5.10 How do you prefer to receive information about pregnancy and AMC?

- - Appointment at the outpatient clinic
  - Appointment by phone (with or without seeing each other/video-interactive meeting)
  - Other: … (open answer possible)

5.11 Which topics definitely have to be discussed during a pre-pregnancy counselling? (multiple answers possible)

- - Fertility
  - Medication to be stopped before pregnancy
  - What is the influence of pregnancy and parenthood on my daily functioning with AMC?
  - What is the influence of AMC on my baby’s and my own health?
  - What are the most common difficulties during pregnancy in women with AMC?
  - Possibilities of genetic testing before/during the pregnancy and chance for heredity (chance of affected child)
  - Possibility of 20-week ultrasound during the pregnancy to exclude AMC
  - Information on labour and preparing your “Birth plan” (plan including amongst others location of delivery, at what gestational age, vaginal delivery or caesarean section, pain relief, presence of which professional caregivers)
  - Breastfeeding and possibility of referral to an occupational therapist for advices
  - Possibilities to deploy help to maximize independency around the pregnancy (assisted by general practitioner, rehabilitation doctor or surrounding non-professional caregivers)
  - Other: … (open answer possible)

5.12 Do you think it would be useful to offer a pre-pregnancy counselling as standard care to all women with AMC?

- - Yes
  - No

5.13 What are your thoughts about fertility in women with AMC compared to other women without AMC?

- - Reduced fertility
  - Equal fertility
  - I don’t know

5.14 Do you have any additional ideas about the “ideal” pre-pregnancy counselling? (when, how, where and with whom, …?): … (open answer possible)

**Information about pregnancy and delivery**

6.1 Answer the next statement: During pregnancy I feel supported if my gynaecologist collaborates with the doctor who treats me for AMC when I’m not pregnant (for example rehabilitation doctor):

- - Yes, early in pregnancy
  - Yes, before the delivery
  - No
  - Other: … (open answer possible)

6.2 Which healthcare provider(s) do you expect to be in need (in your case) during your pregnancy other than obstetric caregiver? (multiple answers possible)

- - General practitioner
  - Rehabilitation doctor
  - Neurologist
  - Orthopaedic surgeon
  - Social worker
  - Psychologist
  - Anesthesiologist
  - Clinical geneticist
  - Paediatrician
  - Multidisciplinary team with tailored care for my situation with AMC: … (open answer possible)
  - Other: … (open answer possible)

6.3 The “Birth plan” helps you and the professional caregivers at your delivery to memorize special wishes and needs during the delivery. Which aspects do you want/would you have wanted to discuss with your gynaecologist/obstetric caregiver and record in your “Birth plan”? (multiple answers possible)

- - Location of labour (for example at home or in a hospital)
  - Mode of delivery: spontaneous vaginal delivery
  - Mode of delivery: induced labour
  - Mode of delivery: caesarean section
  - Discussion about at which pregnancy week
  - Possibility of pain relief (for example epidural)
  - Which professional healthcare providers will be present during the labour
  - If vaginal delivery: position during delivery
  - Breast and formula feeding
  - Extra care after birth

6.4 How do you prefer to deliver the baby adapted to your AMC?

- - Vaginally
  - Caesarean section
  - No preference, but I would prefer to make the choice during my labour
  - Not applicable, because I don’t have the wish to become pregnant
  - Other: … (open answer possible)

6.5 A “Birth plan” has to be adapted often due to certain developments during pregnancy. When would you prefer to discuss your “birth plan” with your healthcare providers? (multiple answers possible)

- - Before pregnancy, for example during a pre-pregnancy counselling
  - At the beginning of the pregnancy (before week 20)
  - Half way the pregnancy (around week 20)
  - At the end of the pregnancy (around week 36)
  - When the contractions start
  - After a change in circumstances, for example when your baby is in breech position
  - Not applicable
  - Other: … (open answer possible)

6.6 If you have been pregnant before: did you prepare a “Birth plan” together with your gynaecologist?

- - I never delivered
  - No, I did not prepare a “Birth plan” with the gynaecologist during my prior pregnancy
  - Yes, therefore my gynaecologist and I were better prepared for labour
  - Yes, but we didn’t follow the “Birth plan” due to known reasons
  - Yes, but we didn’t follow the “Birth plan” due to unknown reasons

6.7 Statement: I would appreciate the possibility to have a separate consultation with the anesthesiologist.
Explanation: an anesthesiologist is a doctor who is involved in giving medicines during an operation (for example, during a caesarean section, inserting infusion, inserting regional analgesia, taking care of optimal position during the operation). An anesthesiologist can also advice on and perform pain relief during childbirth.

- - Yes
  - No
  - Neutral
  - Not applicable

6.8 If you had a delivery: did consultation with an anesthesiologist occur during that pregnancy?

- - Yes
  - No
  - Not applicable

**Information about period after birth**

Please indicate whether you agree or not with the following statements relating to the period after the delivery:

7.1 I think that I don’t need extra help of my own network (family, friends and healthcare providers) during physical care of myself and my child

- - Agree
  - Not agree

7.2 I think that I don’t need extra help for households after my delivery

- - Agree
  - Not agree

7.3 I think that I don’t need extra aid(s) for beastfeeding my child

- - Agree
  - Not agree

7.4 I think that I don’t need extra aid(s) for formula feeding my child

- - Agree
  - Not agree

7.5 I think that I don’t need extra aid(s) to care my child

- - Agree
  - Not agree

**Your opinion and advice**

8.1 We would like to thank you again for completing this questionnaire. It is possible that you have missed certain aspects or questions in this questionnaire. Share your ideas and suggestions with us in the box. Also website addressed with helpful information are welcome. Examples of questions are: “What was the most important/relevant advice you were given, and by whom?”, “What type of advice did you wish someone had given you during your entire pre-pregnancy/pregnancy-delivery journey?” … (open answer possible)
