# Supplementary material for: Arthrogryposis Multiplex Congenita (AMC) and counselling before and during pregnancy: a questionnaire study
Source: Orphanet J Rare Dis. 2025 Jul 26;20:378. doi: 10.1186/s13023-025-03913-y (PMC12297761; doi:10.1186/s13023-025-03913-y)
Supplement: Supplementary file 2 — Additional file 2. [file 13023_2025_3913_MOESM2_ESM.docx]

**Additional File Tables 2-8. Results Survey AMC and pregnancy in 2024**

| Question number | Responses | Number / completed questions (percentage) |
| --- | --- | --- |
| 1.1 | Age  16-20 years old 21-25 years old 26-30 years old 31-35 years old 36-40 years old 41-45 years old 46-50 years old 51-55 years old 56-60 years old 61-65 years old 66-70 years old 71-75 years old 76-80 years old 81+ years old | 2/53 (3.8%) 3/53 (5.7%) 5/53 (9.4%) 8/53 (15.1%) 9/53 (17.0%) 6/63 (11.3%) 4/53 (7.5%) 9/53 (17.0%) 3/53 (5.7%) 3/53 (5.7%) 0 1/53 (1.9%) 0 0 |
| 1.2 | Country of living  Spain United States  The Netherlands  United Kingdom Canada France Switzerland Open answer: Norway Open answer: Italy Open answer: New Zealand | 27/53 (50.9%) 10/53 (18.9%) 6/53 (11.3%) 4/53 (7.5%) 2/53 (3.8%) 1/53 (1.9%) 0/53 1/53 (1.9%) 1/53 (1.9%) 1/53 (1.9%) |

***Additional File Table 2. AMC and pregnancy, survey among 53 women with AMC in 2024, General background***

| Question number | Responses | Number / completed questions (percentage) |
| --- | --- | --- |
| 2.1 | Location joint contractures  Upper limb(s) Lower limb(s) Spine and/or neck (inclusive scoliosis, a curvature in the spine) Jaw Other | 40/53 (75.5%) 49/53 (92.5%) 31/53 (58.5%)  10/53 (18.9%) 0 |
|  | Combinations of involved joints  Upper and lower limbs, spine and or neck Upper and lower limbs Lower limbs, spine and or neck Upper and lower limbs, and jaw Lower limbs Upper and lower limbs, spine and or neck, and jaw Lower limbs, and jaw Spine and or neck, and jaw Lower limbs, spine and or neck, and jaw | 22/53 (41.5%) 14/53 (26.4%) 5/53 (9.4%) 3/53 (5.7%) 3/53 (5.7%) 2/53 (3.8%)  2/53 (3.8%) 1/53 (1.9%) 1/53 (1.9%) |
| 2.2 | Independency in use of lower limbs  Walking independently without devices Walking independently with braces Walking partly independently, partly with aid(s), such as sticks/crutches/canes/walker Walking partly independently, partly with wheelchair Mainly with wheelchair Open answer: help from parents Open answer: special footwear Open answer: walking with aids and ambulatory wheelchair use) | 24/53 (45.3%) 4/53 (7.5%) 2/53 (3.8%)  6/53 (11.3%)  14/53 (26.4%) 1/53 (1.9%) 1/53 (1.9%) 1/53 (1.9%) |
| 2.3 | Independency in use of arms for daily activities such as dressing and physical care  Independent Fully dependent Partially independent | 24/53 (45.3%) 9/53 (17.0%) 20/53 (37.7%) |
| 2.4 | Independency in use of arms for daily activities such as eating  Independent Fully dependent Partially independent | 37/53 (69.8%) 2/53 (3.8%) 14/53 (26.4%) |
| 2.5 | Independency in use of arms for daily activities such as housekeeping/household  Independent Fully dependent Partially independent | 16/53 (30.2%) 12/53 (22.6%) 25/53 (47.2%) |
| 2.6 | Independency in use of arms for daily activities such as using a computer  Independent Fully dependent Partially independent | 45/53 (84.9%) 1/53 (1.9%) 7/53 (13.2%) |
| 2.7 | Independency in use of arms for daily activities such as cycling  Independent Fully dependent Partially independent | 21/53 (39.6%) 23/53 (43.4%) 9/53 (17.0%) |
| 2.8 | Independency in use of arms for daily activities such as driving  Independent Fully dependent Partially independent | 35/53 (66.0%) 12/53 (22.6%) 6/53 (11.3%) |
| 2.9 | Previous surgery in upper limbs (from shoulders to fingers)  Yes No | 16/53 (30.2%) 37/53 (69.8%) |
| 2.9.1 | Area of surgery in upper limbs  Shoulder(s) Elbow(s) Wrist(s) Finger(s) | 4/16 (25.0%) 4/16 (25.0%) 12/16 (75.0%) 8/16 (50.0%) |
| 2.9.2 | Number of operation(s) in upper limb(s)  1 2 3 4 5 >5 | 3/16 (18.8%) 2/16 (12.5%) 3/16 (18.8%) 2/16 (12.5%) 2/16 (12.5%) 4/16 (25%) |
| 2.10 | Previous surgery in lower limbs (from hips to feet)  Yes No | 46/53 (86.8%) 7/53 (13.2%) |
| 2.10.1 | Area of surgery in lower limbs  Hip(s) Knee(s) Ankle(s) Feet | 19/46 (41.3%) 26/46 (56.5%) 33/46 (71.7%) 37/46 (80.4%) |
| 2.10.2 | Number of operation(s) in lower limb(s)  1 2 3 4 5 >5 | 6/46 (13.0%) 2/46 (4.3%) 6/46 (13.0%) 8/46 (17.4%) 1/46 (2.2%) 23/46 (50%) |
| 2.11 | Previous surgery in spine and/or neck  Yes No | 6/53 (11.3%) 47/53 (88.7%) |
| 2.11.1 | Number of operation(s) in neck and/or spine  1 2 3 4 5 >5 | 5/6 (83.3%) 0 1/6 (16.7%) 0 0 0 |
|  | See supplementary figure 1. |  |
| 2.13 | See supplementary figure 1. |  |
| 2.14 | Underlying cause for AMC known  Yes  No | 14/53 (26.4%)  39/53 (73.6%) |
| 2.14.1 | Underlying cause of AMC (open answers, multiple causes per person described)  Larsen syndrome Hereditary Amyoplasia Malformation of uterus Loss/lack of amniotic fluid Reduced fetal movement, e.g. twin pregnancy Aging Medicinal/iatrogenic e.g. Thalidomide | 1/14 (7.1%) 5/14 (35.7%) 1/14 (7.1%) 1/14 (7.1%) 3/14 (21.4%) 2/14 (14.3%) 1/14 (7.1%) 2/14 (14.3%) |
| 2.14.2 | Genetic cause for AMC  Yes No  Unknown | 5/14 (35.7%) 1/14 (7.1%) 8/14 (57.1%) |
| 2.15 | Genetic tests performed to find underlying cause of AMC  Yes No Unknown | 19/53 (35.8%) 27/53 (50.9%) 7/53 (13.2%) |
| 2.15.1 | Year(s) genetic test(s) were performed (multiple answers possible)  1980  1994  1995  2007  2016  2017  2018  2019  2020 2021  2022 2023 Unknown | 1/19 (5.3%) 1/19 (5.3%) 1/19 (5.3%) 1/19 (5.3%) 1/19 (5.3%) 2/19 (10.5%) 1/19 (5.3%) 1/19 (5.3%) 0/19  3/19 (15.8%) 1/19 (5.3%) 4/19 (21.1%) 5/19 (26.3%) |
| 2.16 | Open for genetic counselling in regard to new possibilities in genetic testing*  Yes No I don’t know | 39/53 (73.6%) 5/53 (9.4%) 9/53 (17.0%) |
| 2.16.1 | Arguments to be informed about possible new genetic testing (open answers)  Curiosity, diagnostic uncertainty Family planning, genetic inheritance Research on AMC Unknown | 23/39 (59.0%) 14/39 (35.9%) 1/39 (2.6%) 1/39 (2.6%) |
| 2.16.2 | Arguments to not be informed about possible new genetic testing (open answers)  No longer in fertile phase Already been a mother through egg donation Would not make any difference Tests have already been performed Unknown | 1/5 (20.0%) 1/5 (20.0%) 1/5 (20.0%) 1/5 (20.0%) 1/5 (20.0%) |
| * Further described in theme 3 in results section in text | | |

***Additional File Table 3. AMC and pregnancy, survey among 53 women with AMC in 2024, Medical background***

| Question number | Responses | Number / completed questions (percentage) |
| --- | --- | --- |
| 3.1 | Living situation  Independent, on my own Independent, with partner without children Independent with partner and children Living with parents or family Living with help of other people (daily/frequent support for daily care) Living in a nursing home | 3/53 (5.7%) 12/53 (22.6%) 18/53 (34.0%) 12/53 (22.6%) 8/53 (15.1%)  0 |
| 3.2 | Highest level of education  Primary/elementary school Secondary/high school Vocational education or similar training Bachelor’s Degree Master’s Degree Open answer: college (briefly) Open answer: Doctorate (PhD) Open answer: Education Specialist (Ed.S) Unknown | 4/53 (7.5%) 10/53 (18.9%) 9/53 (17.0%) 16/53 (30.2%) 8/53 (15.1%) 2/53 (3.8%) 2/53 (3.8%) 1/53 (1.9%) 1/53 (1.9%) |
| 3.3 | Currently working  Yes No | 36/53 (67.9%) 17/53 (32.1%) |
| 3.3.1  3.3.2 | Type of employment  Part time Fulltime  Paid Unpaid work/volunteer | 13/36 (36.1%) 23/36 (63.9%)  35/36 (97.2%) 1/36 (2.8%) |
| 3.3.3 | Specification field of employment (open answers)  Healthcare Civil service/ public policy Administrative  Facilities  Technology Others Not specified | 10/36 (27.8%) 4/36 (11.1%) 4/36 (11.1%) 4/36 (11.1%) 3/36 (8.3%) 7/36 (19.4%) 4/36 (5.6%) |
| 3.3.4 | Reasons for being unemployed  Student Retired Open answer: not able to work due to limitations of AMC Open answer: taking care of children  Open answer: did not find a job yet | 4/17 (23.5%) 5/17 (29.4%) 5/17 (29.4%)  2/17 (11.8%) 1/17 (5.9%) |
| 3.4 | Partner  Yes No | 37/53 (69.8%) 16/53 (30.2%) |
| 3.5 | Energy for hobbies and sports  Yes No  Not at this moment, but did have in the past | 31/53 (58.5%) 5/53 (9.4%) 17/53 (32.1%) |
| 3.5.1 | Specification type of hobbies and sports (open answers)  Physical activities/sports Entertainment Creative activities Reading and writing  Games Not specified | 22/31 (71.0%) 12/31 (38.7%) 7/31 (22.6%) 7/31 (22.6%) 3/31 (9.7%) 1/31 (3.2%) |
| 3.6 | See supplementary figure 2. |  |

**Additional File Table 4 AMC and pregnancy, survey among 53 women with AMC in 2024, Social life, education and work**

| Question number | Responses | Number / completed questions (percentage) |
| --- | --- | --- |
| 4.1 | Ever been pregnant (including miscarriages, termination of pregnancies and/or stillbirths)  Yes No | 34/53 (64.2%) 19/53 (35.8) |
| 4.1.1 | Reasons for never been pregnant  Own choice Advice healthcare provider Wish to get pregnant but never full-term pregnancy and no miscarriages Not able to have intercourse Not able to use insemination support | 15/19 (78.9%)  2/19 (10.5%)  1/19 (5.3%)   1/19 (5.3%)  0 |
| 4.1.2 | Number of pregnancies  1 2 3 4 5 >5 | 11/34 (32.4%) 14/34 (41.2%) 6/34 (17.6%) 1/34 (2.9%) 2/34 (5.9%) 0 |
| 4.1.3 | Number of alive children given birth to  1 2 3 4 5 >5 | 12/26 (46.2%) 11/26 (42.3%) 2/26 (7.7%) 0  1/26 (3.8%) 0 |
| 4.1.3.1 | Healthy child(ren)  Yes No | 45/45 (100.0%) 0 |
| 4.1.3.2 | Child(ren) affected with AMC  Yes No | 0 45/45 (100.0%) |
| 4.1.3.3 | Child(ren) affected with other diseases  Yes No | 9/26 (34.6%) 17/26 (65.4%) |
| 4.1.4 | Mode of delivery  Vaginal Caesarean section (C-section) | 15/45 (33.3%) 30/45 (66.7%) |
| 4.1.4.1 | Number of vaginal births per woman  1 2 3 4 5 >5 | 6/15 (40.0%) 1/15 (6.7%) 1/15 (6.7%) 1/15 (6.7%) 0 0 |
| 4.1.4.2 | Gestational age at vaginal birth in weeks (open answers)  32  33  34  37  38  39  40  42  Preterm birth A term birth | 1/15 (6.7%) 1/15 (6.7%) 1/15 (6.7%) 3/15 (20%) 3/15 (20%) 1/15 (6.7%) 4/15 (26.7%) 1/15 (6.7%)  3/15 (20.0%) 12/15 (80.0%) |
| 4.1.4.3 | Complications vaginal birth (multiple answers possible)  Uneventful without complications Assisted with vacuum cup Difficulties during application epidural Assisted with forceps Difficulties during inserting IV drip Open answer: episiotomy | 10/15 (66.7%) 2/15 (13.3%) 2/15 (13.3%) 1/15 (6.7%) 0/15 1/15 (6.7%) |
| 4.1.4.4 | Number of C-sections per woman  1 2 3 4 5 >5 | 10/30 (33.3%) 7/30 (23.3%) 2/30 (6.7%) 0 0 0 |
| 4.1.4.5 | Gestational age at C-section (open answers)  32  34  36  37  38  39  40  41  “Full term”  Not specified  Preterm birth A term birth  Unknown | 1/30 (3.3%) 2/30 (6.7%) 1/30 (3.3%) 1/30 (3.3%) 10/30 (33.3%) 4/30 (13.3%) 4/30 (13.3%) 1/30 (3.3%) 3/30 (10.0%) 3/30 (10.0%) 4/30 (13.3%) 22/30 (73.3%) 3/30 (10.0%) |
| 4.1.4.6 | C-section planned  Yes, early in pregnancy Yes, before delivery  No Unknown | 18/30 (60.0%) 5/30 (16.7%) 2/30 (6.7%) 5/30 (6.7%) |
| 4.1.4.7 | Complications C-section (multiple answers possible)  No complications There were difficulties/complications during the caesarean section(s)  Difficulties during application epidural or spinal  Difficulties with positioning on delivery table  Difficulties during inserting IV drip  Difficulties during intubation | 27/30 (90.0%)  2/30 (6.7%)  3/30 (10.0%)  0/30  0/30 |
| 4.1.5 | Location delivery per woman  Hospital (midwife assisted) Hospital (doctor assisted) Hospital (midwife and doctor assisted) Home (midwife or general practitioner assisted) | 3/26 (11.5%) 18/26 (69.2%) 5/26 (19.2%) 0 |
| 4.1.6 | Course of pregnancy leading to decision to not become pregnant again  Yes  No Don’t know yet | 12/34 (35.3%) 21/34 (61.8%) 1/34 (2.9%) |
| 4.1.7 | Extra help from own network (friends or family)  During pregnancy After pregnancy No extra help | 14/34 (41.2%) 17/34 (50.0%) 13/34 (38.2%) |
| 4.1.8 | Extra help for housekeeping/household  During pregnancy After pregnancy No extra help | 12/34 (35.3%) 18/34 (52.9%) 15/34 (44.1%) |
| 4.1.9 | More assistance required with self-care  During pregnancy After pregnancy No extra help | 13/34 (38.2%) 11/34 (32.4%) 18/34 (52.9%) |
| 4.1.10 | Adaptions/help for breastfeeding  Yes No | 8/26 (30.8%) 18/26 (69.2%) |
| 4.1.11 | Adaptions/help for formula feeding  Yes No | 6/26 (23.1%) 20/26 (76.9%) |
| 4.1.12 | Adaptions/help for childcare (e.g cleaning)  Yes No | 12/26 (46.2%) 14/26 (53.8%) |
| 4.1.13 | Change in mobility during or after pregnancy (open answers) per women (multiple answers possible)  Yes  Due to increase in pain (hips, back, arms, pelvic) Difficulties walking, need to use wheelchair Worsening of joint complaints (arthrosis, osteoarthritis) Other (e.g. fatigue, swelling of legs, weakness)  No | 18/26 (69.2%)  7/26 (26.9%)  8/26 (30.8%)  4/26 (15.4%)  4/26 (15.4%)  8/26 (30.8%) |

***Additional File Table 5. AMC and pregnancy, survey among 53 women with AMC in 2024, Experience during earlier pregnancies.***

| Question number | Responses | Number / completed questions (percentage) |
| --- | --- | --- |
| 5.1 | Preferred way to be informed about pregnancy and AMC  Other patients with AMC Internet/website Meeting with presentations by healthcare providers Via own healthcare provider by advise consultation  Discussion forum on internet Patient support groups Information leaflets Don’t want to be informed Magazines Other | 31/52 (59.6%) 28/52 (54.8%) 27/52 (51.9%)  24/52 (46.2%)  23/52 (44.2%) 18/52 (34.6%) 15/52 (28.8%) 7/52 (13.4%) 5/52 (9.6%) 0 |
| 5.2 | Talked with healthcare providers about: wish to have children  Yes Yes, but healthcare providers couldn’t answer questions No, because healthcare providers never had time  No, because I felt it’s difficult to talk about this topic Open answer: did not feel the need to  Open answer: diagnosis AMC was unknown Open answer: topic has never been addressed | 19/52 (36.5%) 11/52 (21.2%)  2/52 (3.8%) 11/52 (21.2%)  7/52 (13.5%) 1/52 (1.9%) 1/52 (1.9%) |
| 5.3 | Talked with healthcare providers about: sexuality  Yes Yes, but healthcare providers couldn’t answer questions No, because healthcare providers never had time  No, because I felt it’s difficult to talk about this topic Open answer: did not feel the need to  Open answer: topic has never been addressed | 16/51 (31.4%) 3/51 (5.9%)  6/51 (11.8%) 18/51 (35.3%)  9/51 (17.6%) 2/51 (3.9%) |
| 5.4 | Sex life (in fertile phase)  Normal, no extra pain  Normal but some extra pain Nearly normal but very painful Severely restricted by pain Nearly absent because of pain Pain prevents any sex life at all Not applicable | 31/51 (60.8%) 12/51 (23.5%) 1/51 (2.0%) 1/51 (2.0%) 0 0  6/51 (11.7%) |
| 5.5 | Talked with healthcare providers about: fertility  Yes Yes, but healthcare providers couldn’t answer questions No, because healthcare providers never had time  No, because I felt it’s difficult to talk about this topic Open answer: did not feel the need to  Open answer: diagnosis AMC was unknown  Open answer: topic has never been addressed | 20/51 (39.2%) 5/51 (9.8%)  4/51 (7.8%) 10/51 (19.6%)  9/51 (17.6%) 1/51 (2.0%) 2/51 (3.9%) |
| 5.6 | Heard of pre-pregnancy counselling  Yes No | 25/51 (49.0%) 26/51 (51.0%) |
| 5.7 | Preferred age to get information about fertility and future child wish  15-16 years old 17-18 years old >18 years old Open answer: Age-appropriate information from nursery or primary school onwards  Open answer: not specified | 2/51 (3.9%) 4/51 (7.8%) 32/51 (62.7%)  2/51 (3.9%) 11/51 (21.6%) |
| 5.8 | Preferred company during pre-pregnancy counselling  Alone (without anyone else) Together with my partner Together with my parents Don’t know Open answer: not specified | 14/50 (28.0%) 26/50 (52.0%) 1/50 (2.0%) 1/50 (2.0%) 8/50 (16.0%) |
| 5.9 | Preferred healthcare provider to give pre-pregnancy counselling  General practitioner Gynaecologist Anesthesiologist Rehabilitation doctor/physiatrist Midwife Neurologist Clinical geneticist Combination of doctors  Open answer: trauma surgeon Open answer: not specified | 17/50 (34.0%) 39/50 (78.0%) 9/50 (18.0%) 5/50 (10.0%) 13/50 (26.0%) 7/50 (14.0%) 20/50 (40.0%) 11/50 (22.0%) 2/50 (4.0%) 7/50 (14.0%) |
| 5.10 | Preferred appointment for pre-pregnancy counselling  Outpatient clinic By phone (with or without seeing each other/video-interactive meeting) Open answer: in hospital Open answer: at mutual insurance company Open answer: not specified | 27/50 (54.0%) 11/50 (22.0%)  2/50 (4.0%) 1/50 (2.0%) 9/50 (18.0%) |
| 5.11 | Preferred topics during pre-pregnancy counselling  Fertility Medication to be stopped before pregnancy Influence of pregnancy and parenthood on daily functioning with AMC Influence of AMC on baby’s and own health Most common difficulties during pregnancy in women with AMC Possibilities of genetic testing before/during pregnancy and chance for heredity  Possibility of 20-week ultrasound during pregnancy to exclude AMC Information on labour and preparing “Birth plan” Breastfeeding and possibility of referral to occupational therapist for advice Possibilities to deploy help to maximize the independency around pregnancy Open answer: emotions that may arise, maternal mental health Open answer: guidance and public subsidies for IVF treatment | 33/50 (66.0%) 29/50 (58.0%) 37/50 (74.0%)  37/50 (74.0%) 39/50 (78.0%)  37/50 (74.0%)  29/50 (58.0%)  35/50 (70.0%) 31/50 (62.0%)  32/50 (64.0%)  1/50 (2.0%)  1/50 (2.0%) |
| 5.12 | Pre-pregnancy counselling useful as standard care to all women with AMC  Yes No | 45/47 (95.7%) 2/47 (4.3%) |
| 5.13 | Thoughts on fertility in women with AMC compared to other women without AMC  Reduced fertility Equal fertility  I don’t know | 5/47 (10.6%) 20/47 (42.6%) 22/47 (46.8%) |

***Additional File Table 6. AMC and pregnancy, survey among 53 women with AMC in 2024, Information before pregnancy***

| Question number | Responses | Number / completed questions (percentage) |
| --- | --- | --- |
| 6.1 | Feel supported if during pregnancy gynaecologist collaborates with doctor for AMC  Yes, early in pregnancy Yes, before the delivery  No Open answer: Don’t have a doctor for AMC | 26/47 (55.3%) 18/47 (38.3%) 11/47 (23.4%) 1/47 (2.1%) |
| 6.2 | Expected healthcare provider(s) to be in need during pregnancy (other than obstetric caregiver)  General practitioner Rehabilitation doctor  Neurologist Orthopaedic surgeon Social worker  Psychologist Anesthesiologist Clinical geneticist Paediatrician Multidisciplinary team with tailored care for own situation with AMC  Open answer: Occupational therapist | 25/47 (53.2%) 18/47 (38.3%)  7/47 (14.9%) 11/47 (23.4%) 7/47 (14.9%)  10/47 (21.3%) 23/47 (48.9%) 18/47 (38.3%) 18/47 (38.3%) 18/47 (38.3%)  1/47 (2.1%) |
| 6.3 | Topics birth plan  Location of labour Mode of delivery: spontaneous vaginal delivery Mode of delivery: induced labour Mode of delivery: caesarean section Discussion about at which pregnancy week Possibility for pain relief Which healthcare provider present during labour If vaginal delivery: position during delivery Breast and formula feeding  Extra care after birth | 29/46 (63.0%) 21/46 (45.7%) 15/46 (32.6%) 31/46 (67.4%) 22/46 (47.8%) 31/46 (67.4%) 28/46 (60.9%) 25/46 (54.3%) 30/46 (65.2%) 36/46 (78.3%) |
| 6.4 | Preferred mode of delivery adapted to your AMC  Vaginally Caesarean section No preference, but would prefer to make choice during labour Not applicable, don’t have the wish to become pregnant Open answers: No preference, but would prefer to discuss the options in consultation and receive information about what is possible and can be facilitated (e.g. adaption of delivery table) | 12/46 (26.1%) 19/46 (41.3%) 7/46 (15.2%)  4/46 (8.7%)  4/46 (8.7%) |
| 6.5 | Preferred moment to discuss birth plan with healthcare providers  Before pregnancy At the beginning of pregnancy (before week 20) Half way the pregnancy (around week 20) At the end of the pregnancy (around week 36) When contractions start After a change in circumstances (e.g. breech position) Not applicable  Other | 15/46 (32.6%) 22/46 (47.8%) 23/46 (50%) 14/46 (30.4%) 4/46 (8.7%) 15/46 (32.6%) 5/46 (10.9%)  0 |
| 6.6 | Prepared birth plan with gynaecologist during prior pregnancy  I never delivered  No, did not prepare birth plan with gynaecologist Yes, therefore gynaecologist and I were better prepared for labour Yes, but didn’t follow birth plan due to known reasons Yes, but didn’t follow birth plan due to unknown reasons | 21/46 (45.7%) 13/46 (28.3%) 9/46 (19.6%)  3/46 (6.5%)  0 |
| 6.7 | Appreciate possibility to have separate consultation with anesthesiologist  Yes No Neutral Not applicable | 33/46 (71.7%) 1/46 (2.2%) 9/46 (19.6%) 3/46 (6.5%) |
| 6.8 | Consultation with anesthesiologist during prior pregnancy  Yes No Not applicable | 11/46 (23.9%) 15/46 (32.6%) 20/46 (43.5%) |

***Additional File Table 7. AMC and pregnancy, survey among 53 women with AMC in 2024, Information about pregnancy and delivery.***

| Question number | Responses | Number / completed questions (percentage) |
| --- | --- | --- |
| 7.1 | Help of own network during physical care of myself and child  Agree Not agree | 33/46 (71.7%) 13/46 (28.3%) |
| 7.2 | Help for households after delivery  Agree Not agree | 41/46 (89.1%) 5/46 (10.9%) |
| 7.3 | Aid(s) for breastfeeding  Agree Not agree | 24/46 (52.2%) 22/46 (47.8%) |
| 7.4 | Aid(s) for formula feeding  Agree Not agree | 23/46 (50.0%) 23/46 (50.0%) |
| 7.5 | Aid(s) to care for child  Agree Not agree | 26/46 (56.5%) 20/46 (43.5%) |

***Additional File Table 8. AMC and pregnancy, survey among 53 women with AMC in 2024, Information after delivery.***

| Participants (n=70) | Ever been pregnant yes/no (filled in by all 53 women that was presented this question) | Advices given by women with AMC that have experienced pregnancy (filled in by 31/34 women that was presented this question) | Ideal pre-pregnancy counselling  (filled in by 44/47 women that was presented this question)  No additional ideas n=25 | Opinions (filled in by 25/46 women that was presented this question)  No additional comments n=4  Expressing gratitude and contentment towards the study n=9  Requesting information sources n=3 |
| --- | --- | --- | --- | --- |
| 1 | No |  | No |  |
| 2 | Yes | Assist us in finding our way to hold, breastfeed/formula feed  and change our kids.  This can mean using feet to change a diaper, or even the mouth, using the mouth or chin to lift and position the baby or the need to have our kids placed on/next to us by our partners or personal care assistants.  Also, understand that some don't want staff intruding on that special bonding time. But there's safety in having medical doctors who have an understanding of us moving differently than non-disabled parents. | I think that information about this topic should've come from the doctor that I have most contact and connection with,  and possibilities should've be presented to me during my teens. | If possible, I would love if healthcare providers had information of sources where one can read other AMCers' pregnancy and birthing experiences, through online forums or research forms like this |
| 3 |  |  |  |  |
| 4 |  |  |  |  |
| 5 | Yes | N/A |  |  |
| 6 | Yes | Please listen to your patient. She knows her body better than anyone. | Limitations should be discussed  and to explore the possible emotions that arise after the birth.  Someone who has knowledge of AMC is vital  I would have loved to talk to another AMCer who already had given birth themselves. That option was not available to me at the time. Peer contact with fellow AMCer that experienced pregnancy was not available during own pregnancy. |  |
| 7 | No |  | No |  |
| 8 |  |  |  |  |
| 9 | Yes | Don’t judge someone on their ability to carry a child just because of a physical disability. Not all bodies are the same, prevent ableism  treat us like every other parent. | Perhaps something online would benefit the most. |  |
| 10 | Yes | It was helpful to have additional ultrasounds to identify if there were any contractures.  Not enough gynecologists and obstetricians have accessible offices and my OB expressed anxiety over treating me due to complications. The anesthesiologist was fantastic and was prepared for my needs. | It will be unlikely that OB/GYN's will have understanding of these needs.  It would be helpful for a virtual visit one on one with patient's with the offer of consultation with the patient's OB/GYN as this may not be in their skillset. | My first delivery was an emergency c-section. My second was planned c-section. I felt there were obvious discriminatory experiences during my second. They questioned if I wanted to "tie my tubes" after my son was born and my husband and son had already left the operating room (that should have been discussed towards the end of the pregnancy- not in the OR), the hospital didn't have an accessible suite available at the hospital and then I was questioned on my parenting care-taking "abilities" when using the bathroom and areas was difficult due to not being accessible, and they sent in a social worker to "question" who would be "home with you and the baby" as they felt "I didn't think this through as it was my first baby" - it was actually my second. Despite these frustrations, I requested to speak with the anesthesiologist prior to delivery so we could discuss my needs around the beginning of my 3rd trimester. It was frustrating to hear my OB say things like "if we don't tie your tubes then understand I will be on vacation if you choose to have another baby." |
| 11 | Yes | Due to extra assistance required for personal care I would suggest that mothers with AMC be given a private room if possible.  I would encourage mums with AMC to accept any extra assistance offered by friends, family & professionals. | I think this survey has mostly touched on those options. Survey touched most options. | You’re welcome, nothing specific comes to mind! |
| 12 | No |  | No |  |
| 13 | Yes | Enjoy your pregnancy | Decide when you want to have children |  |
| 14 | Yes | I currently do not feel well support and consequently have struggled with my mental health - depression and anxiety (which has not previously been an issue). | What to expect? Which professionals to be in contact with throughout pregnancy?  Should be delivered by someone who knows what AMC is. | I am currently 24 +3 weeks pregnant and have found care depends on where you live and the professionals own knowledge and opinions. We started the process of getting support early at 12 weeks but have not felt we have received the support we were expecting. Consultant referrals were made but they have decided not to see us without any communication. I have not felt supported. I am worried about what to expect the further into the pregnancy I get and am not sure how I will manage. I would love any info or signposting to resources you can suggest. |
| 15 | Yes | I had to keep explaining my AMC and how it affected my limbs and their lack of movement.  The doctors were able to deliver without any issues. | None |  |
| 16 |  |  |  |  |
| 17 | No |  | no |  |
| 18 |  |  |  |  |
| 19 | No |  | Women with AMC need to be given the same counseling and information about pregnancy and childbirth as able bodied women. The medical field has a lot of internalized ableism around this and seldom initiated conversations about this with me. | I chose not to have a child partially due to lack of educated doctors around AMC and pregnancy/delivery/infant care. I was fearful of how my body would adapt to pregnancy given my AMC and thus chose never to have children. With better resources, I may have chosen differently. |
| 20 | Yes | Give extra care and allow relaxation when required. Don’t overdo it. | With someone familiar with AMC for sure! | None |
| 21 | Yes | More insight into AMC and disabilities in general. | I don’t but I think this is important. | Ok thank you |
| 22 |  |  |  |  |
| 23 | Yes | Listen to the patient.  Early and often prenatal imaging to determine if AMC is present in the baby.  If yes, follow research suggestions for how to keep baby moving in utero to hopefully lessen severity of contractures. | No add | Thank you for conducting this important research!!Contacting a geneticist who specialized in AMC before pregnancy was so helpful! |
| 24 | No |  |  |  |
| 25 |  |  |  |  |
| 26 | Yes | Don't try vaginally, just go for the c section, you will be considered a high risk pregnancy, but don't be scared it's normal for us, ask for HELP don't be embarrassed of anything, be happy, drink water, exercise what you can because baby is gonna need you, reach out to someone who has been there if you ever feel alone!!! | Have after care help with a pregnancy/parent nurse to help adjust to being home and caring for baby. |  |
| 27 | Yes | Better guidance during pregnancy by doctors with knowledge of AMC. | There should be knowledge on AMC. |  |
| 28 | No |  | With a doctor who can empathize with someone with AMC.  If you want to get pregnant the location etc doesn't matter so much |  |
| 29 | Yes |  | I would just like to publicize amc a bit more. You actually have to explain what AMC is to every doctor yourself first. Always succeed if it is still such an unknown condition. Advice more publications and AMC in general. Amongst others to prevent the need for repeating explaining. |  |
| 30 | No |  |  | I would like to thank you very much for this survey. I have always been very much looking for fellow sufferers and I have not necessarily been able to find them. And this desire has only increased following my desire to have children. |
| 31 |  |  |  |  |
| 32 | No |  | I think it would be nice if there was the possibility to discuss this issue first with a GP and then possibly with a gynaecologist and then look further at the options together. | Not applicable |
| 33 |  |  |  |  |
| 34 | No |  | No |  |
| 35 |  |  |  |  |
| 36 |  |  |  |  |
| 37 |  |  |  |  |
| 38 | Yes | To do gentle sport during pregnancy, to take care of themselves as if it were a high-risk pregnancy, to make sure the baby is as healthy as possible and to enjoy their children.  They should make sure that the baby is as healthy as possible and that they enjoy their children.  The doctor should do free genetic tests in each case of AMC to know what to expect or if there will be consequences for the children.  More care, more vitamins. | No, I think I have explained it well. | Right now I can't think which website is better or necessary, my children are already 28 and 30 years old and I'm a bit far away. But I would like to receive information about this survey. |
| 39 | No |  | They should give us an updated list of all the possible genes involved in AMC during pregnancy so that we could do a very complete genetic test.  They should also explain to us how weight gain (in my case extremely small feet) will influence us - circulatory issues - lower extremities. | The number of surgeries to differentiate between major and minor surgeries.  I would like to ask you to send us a complete list of the possible genes involved in AMC and if you could send us an informative leaflet of what is known about AMC and pregnancy - I am a bit worried about fertility, or pain during sexual intercourse or childbirth. Another issue that worries me a lot is the circulatory issue and venous return, or possible vascular ulcers... as I have very small lower limbs and no movement... Thank you very much. |
| 40 | Yes | To seek counselling and preparation for childbirth,  swim if they can, control their weight as much as possible.  Women to request all tests that their community allows to rule out illnesses in their children.  to attend their midwife's workshops, preparation for breastfeeding with appropriate cushions and breast pumps.  Doctors to train staff for births with women with mobility difficulties. | First find out if she is fertile, possibilities of inheriting the disease, risks and care.  Advised by different professionals such as: gynaecologist, geneticist, midwife, rehabilitated, midwife and those necessary according to the case. | I believe that in my case, as I had AMC, I was referred for follow-up in Hospital, but I did not have any counselling regarding my disease because most of the health workers did not know about it. |
| 41 | Yes | Personalized attention according to needs. | No |  |
| 42 | Yes | Treat normally. | No |  |
| 43 | No |  | I don’t know | . |
| 44 | Yes | That there is more human ? | I have no idea |  |
| 45 | Yes | Adaptation of the healthcare space: tables, examination couches, delivery or operating theatre tables, etc.  Prior genetic counselling.  Health personnel should be sensitized and prepared for the care of people with AMC.  A specific healthcare protocol should be drawn up to find out why people with AMC have so many complications when it is necessary to look for veins to insert lines for medication, analytical tests or sedation, thus avoiding unnecessary suffering. | No | It would be very necessary to have a multidisciplinary team adaptations and resources for people with reduced mobility within the health sector,  training and information for health professionals with regard to AMC to guarantee full comprehensive care for the person with AMC. |
| 46 | No |  | No | The situation I have almost always encountered is a direct refusal by health professionals to get pregnant, even though I have not even considered it. |
| 47 | Yes |  |  |  |
| 48 | Yes | More humanity | I have no idea |  |
| 49 | Yes | Having hip problems is not synonymous with having to give birth by caesarean section, I had 1 vaginal birth and another caesarean section but by podalic position. The only thing that the AMC has interfered with is the recovery from the caesarean section and childbirth when doing pelvic recovery exercises. | No |  |
| 50 | Yes | I had a miscarriage in the 3^rd^ month. | Any counselling would already be much better than at present, as there is none.  Ideally it should start from 18+ years of age.  the patient's primary doctors should offer referrals for special gynecological counselling. | Thank you, it has been very interesting. And thank you for your work and interest in our pathology. |
| 51 | Yes | I would tell them to live the experience, which is precious. | No. But in a place that builds trust. |  |
| 52 | No |  |  | Not applicable |
| 53 | Yes | Before the pregnancy I was still able to walk and move with more freedom but not long ceilings.  It would be good if there were more ultrasound scans and tests that are harmless to the baby, not an amniocentesis that puts the baby at risk, and perhaps a free genetic study for those affected and descendants of patients with AMC, which would bring more peace of mind to the mother-to-be and the whole family in particular. | At the gynaecologist with the prescription for the first pill, in order to prepare and include vitamins and minerals as soon as you decide to look for a baby. | The only bad thing about the caesarean was not being able to hold or see my children until 24 hours later. Having been born without any problems they were locked in the incubator during that time. Inhuman and unfair rules for any mother and even more so for the babies. |
| 54 | Yes |  | No | I think it's all great |
| 55 |  |  |  |  |
| 56 |  |  |  |  |
| 57 | Yes | Firstly, the professionals should know what AMC is, so that any doubts the mother-to-be may have can be properly addressed.  And secondly, specific ultrasound scans for the case in question and qualified personnel for this.  To pregnant women with AMC, I would tell them to ask everything they need and not to be afraid of being pestered, to look for a gynecologist who inspires confidence in them so that they are free to ask and ask without feeling bad. And that they have all the information they need. | All of the above marked. | For breastfeeding, I find the website lactancia.org very useful, where you can check which medicines are suitable for breastfeeding and which are not. |
| 58 | No |  |  |  |
| 59 | Yes | To be informed of all problems. | No | I found the questionnaire very interesting |
| 60 | Yes |  |  |  |
| 61 | Yes | Be informed about the particular limitations that each patient with AMC patient may have. | It would be good to have a joint visit of a specialist doctor, a psychologist and a gynaecologist in a health centre. | Many questions I could not answer as they were addressed to future pregnant women. |
| 62 |  |  |  |  |
| 63 | Yes | More humanity. | I have no idea |  |
| 64 | No |  | From primary care: healthcare provider. | Professionals to inform about support products that are on the market to facilitate childcare. |
| 65 | No |  |  |  |
| 66 |  |  |  |  |
| 67 |  |  |  |  |
| 68 | Yes | Everything went well | No | Thank you |
| 69 | No |  | I don’t know | All good |
| 70 | No |  |  |  |
| 71 |  |  |  |  |

***Additional File Table 9. AMC and pregnancy, survey among 53 women with AMC in 2024, individual answers including opinion and advice.***
